# Supplementary material for: The Rustenburg Layered Suite formed as a stack of mush with transient magma chambers
Source: Nat Commun. 2021 Jan 21;12:505. doi: 10.1038/s41467-020-20778-w (PMC7820422; doi:10.1038/s41467-020-20778-w)
Supplement: Supplementary file 1 — Supplementary Information [file 41467_2020_20778_MOESM1_ESM.pdf]

Supplementary information

for

The Rustenburg Layered Suite formed as a stack of mush with transient magma chambers

Zhuo-sen Yao, James E. Mungall*, M. Christopher Jenkins

Department of Earth Sciences, Carleton University, 2115 Herzberg Laboratories, 1125 Colonel By Drive, Ottawa, K1S 5B6, Canada

*Correspondence and requests for materials should be addressed to J.E.M. (JamesMungall@cunet.carleton.ca).

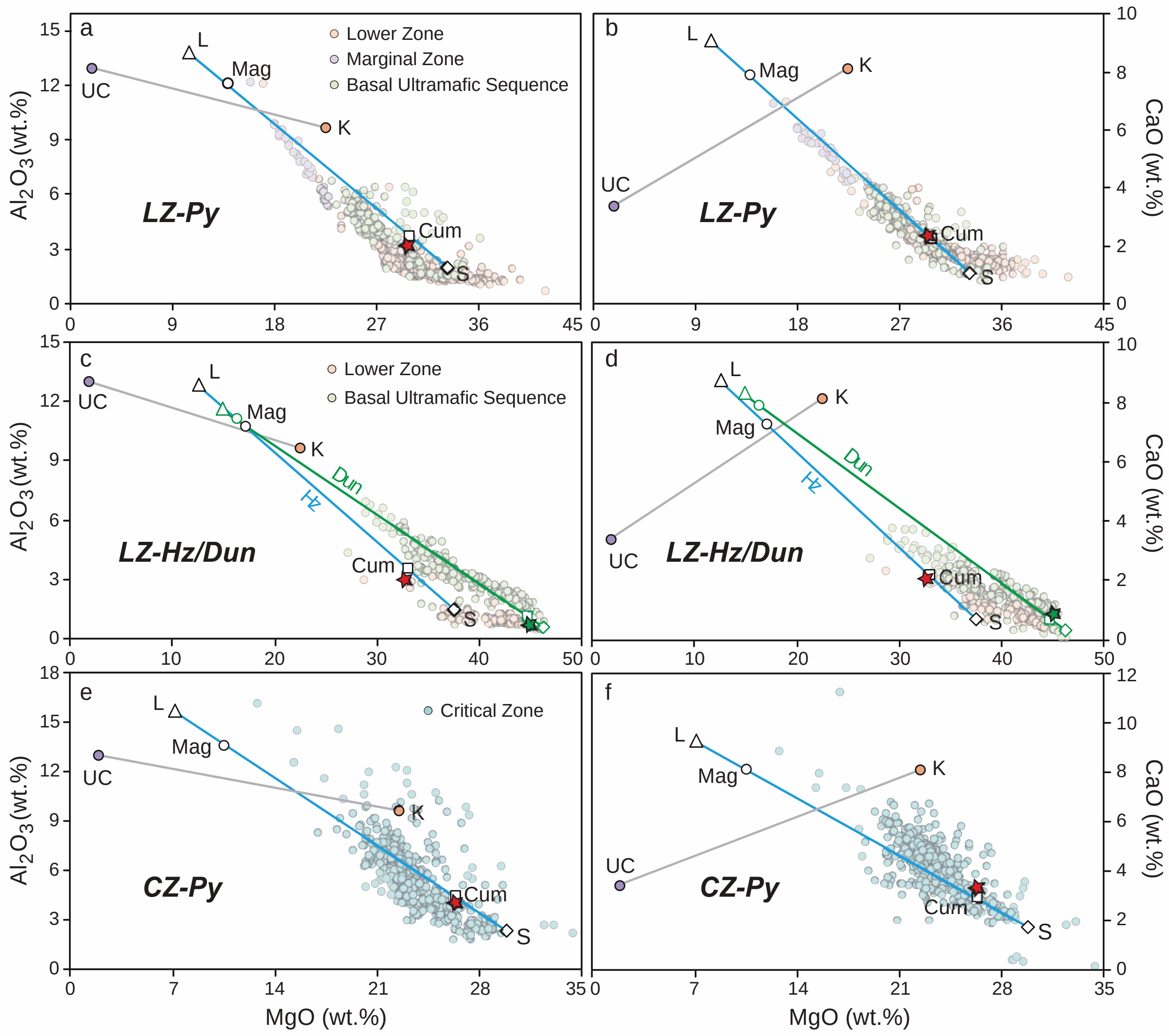

Supplementary Figure 1. Bivariate diagrams for major element whole-rock compositions from the ultramafic cumulates in the Lower Zone (LZ; **panels a to d**) and Critical Zone (CZ; **panels e,f**), and the corresponding model results are included for comparisons. Compositions of komatiite and upper crust are shown as K and UC, respectively. Grey and blue (and green) lines represent the komatiite-crust assimilation trends and the solid-liquid reallocation relationship, respectively. Crystal sorting between the model liquid (L) and solid (S) after assimilation and cooling would generate the observed ultramafic cumulates (Cum) and corresponding phenocryst-bearing magma (Mag) that may form the marginal rocks. The red pentagrams represent the average compositions of corresponding ultramafic cumulates from the representative section of RLS (Fig. 3), meanwhile, a mass of the ultramafic cumulates in RLS have also been collected as the background database. All data are collected from multiple sources cited in the supplementary references[1-8]. Source data are provided as a Source Data file.

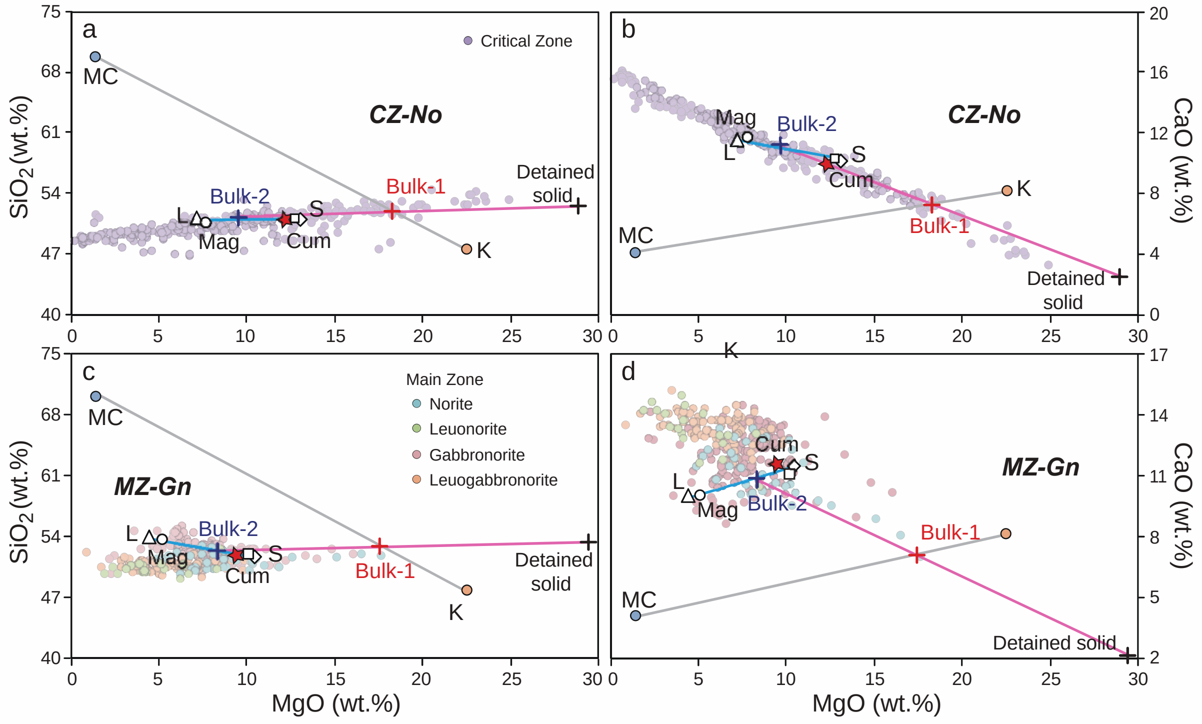

Supplementary Figure 2. Bivariate diagrams for major element whole-rock compositions from the mafic cumulates in the Critical Zone (CZ; a,b) and Main Zone (MZ; c,d), and the corresponding model results are exhibited for comparisons. Compositions of komatiite and middle crust are shown as K and MC, respectively. Grey lines represent the trends of komatiite-crust assimilation occurred in the middle crust, which generated the magmatic systems with the Bulk-1 compositions (red crosses). Most of the solids have been detained in deep (Black crosses), and the remaining supernatant liquid wrapped few solids to form a new magma (Bulk-2 compositions, blue crosses) that rises and undergoes a second batch crystallization as it cools and is emplaced at the level of the RLS to form a mushy microlayer. Crystal sorting between the model liquid (L) and solid (S) after cooling would generate the observed mafic cumulates (Cum) and corresponding phenocryst-bearing magma (Mag) that may form the marginal rocks. The red pentagrams represent the average compositions of corresponding ultramafic cumulates from the representative section of RLS (Fig. 3), meanwhile, a mass of the mafic cumulates in RLS have also been collected as the background database. All data are collected from multiple sources cited in the supplementary references[9-19]. Source data are provided as a Source Data file.

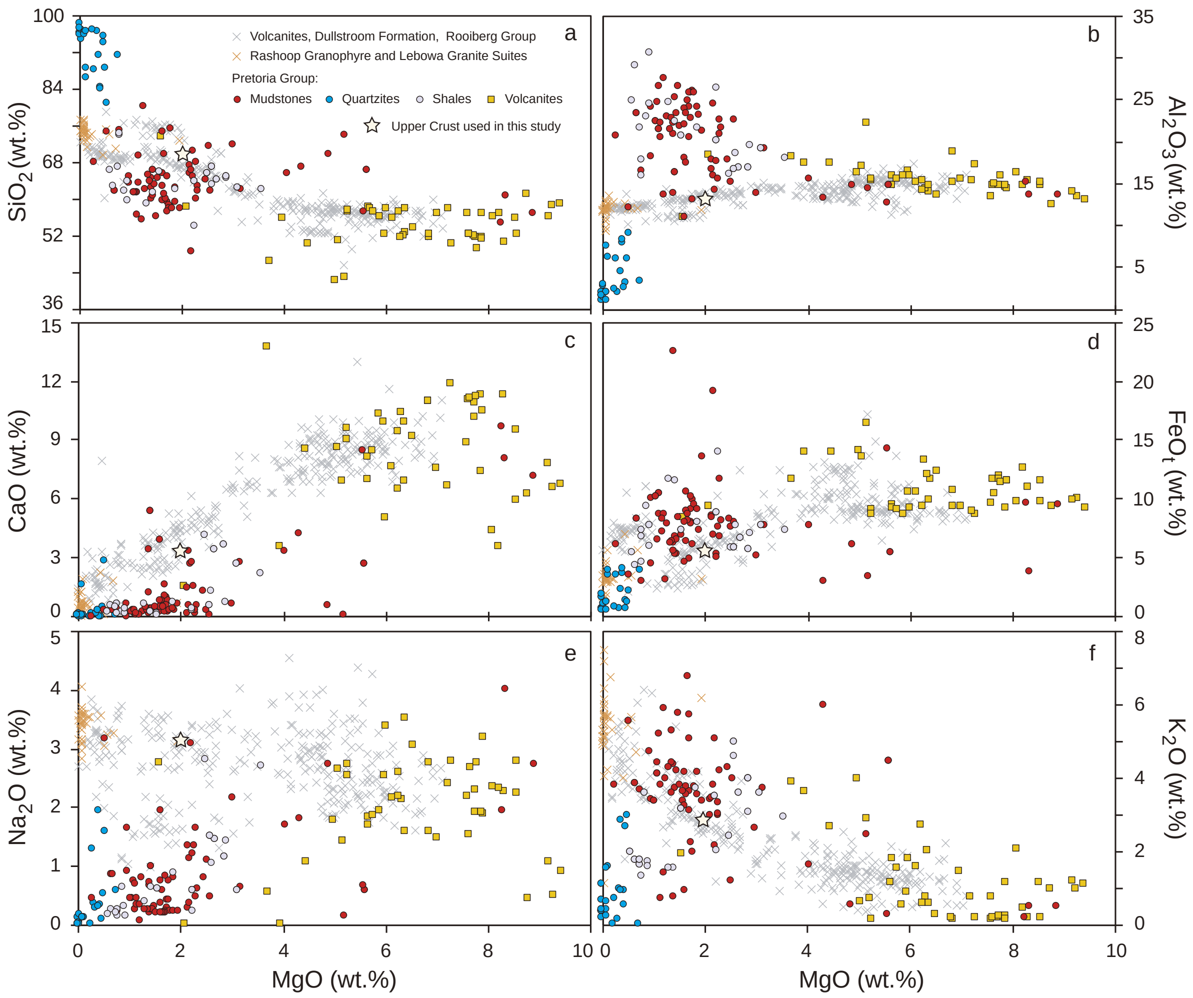

Supplementary Figure 3. MgO against **a-e** SiO_2, Al_2O_3, CaO, FeO_t, Na_2O and K_2O for the complete rock suite of Rooiberg Group, Rashoop Granophyre Suite, Lebowa Granite Suite and Pretoria Group. Because the compositions of sedimentary rocks in Pretoria Group are highly variable, the mixture of them is assumed as the composition of upper-crustal assimilant in this simulation (shown as pentagram), which is also situated on the trends of volcanic rocks from Dullstroom Formation and Rooiberg Group, and additionally similar to the composition of upper crust complied by Gao et al.[20] and Rudnick & Gao[21]. All data are collected from multiple sources cited in the supplementary references[22-49]. Source data are provided as a Source Data file.

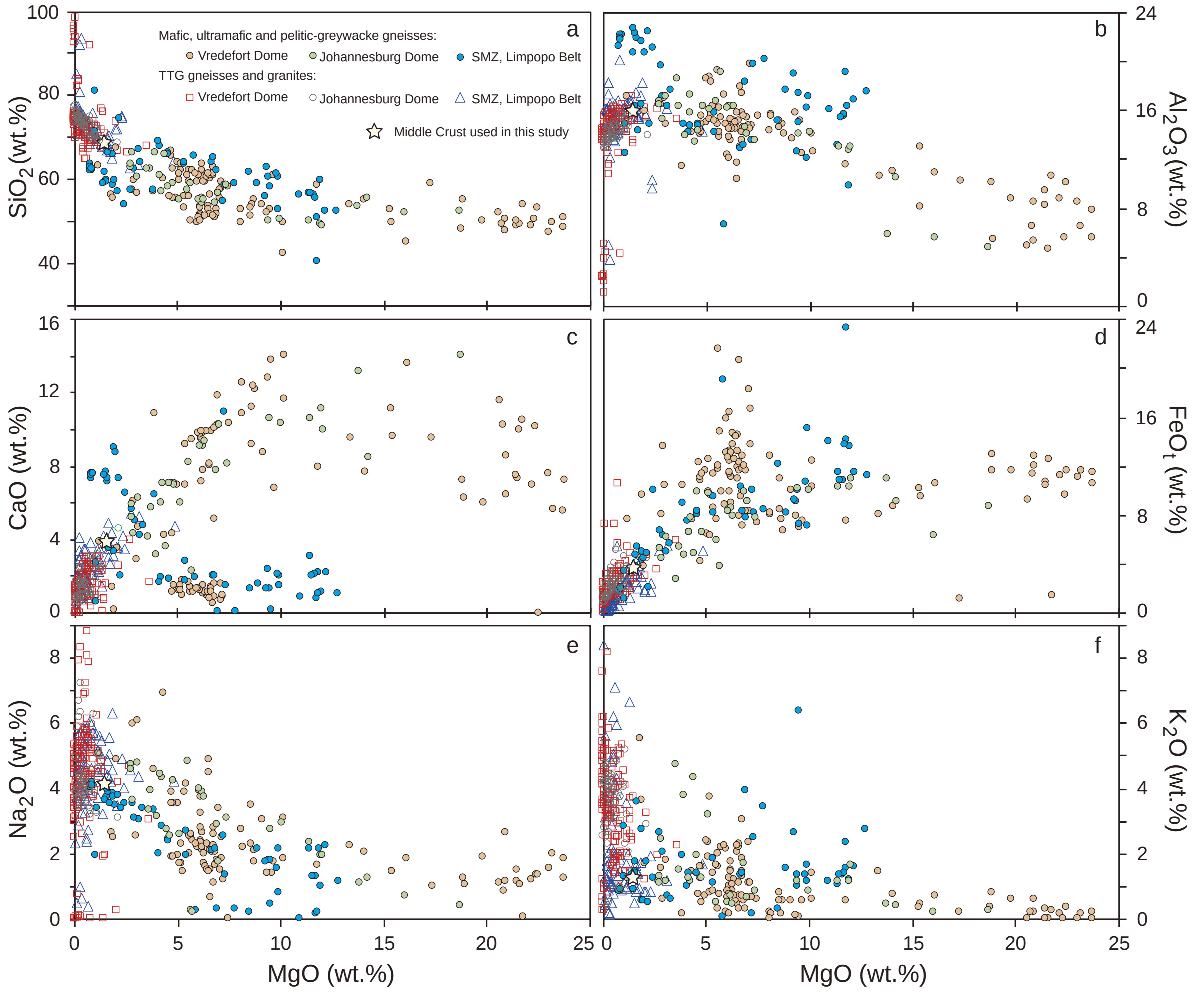

Supplementary Figure 4. MgO against **a-e** SiO_2, Al_2O_3, CaO, FeO_t, Na_2O and K_2O for the potential middle Kaapvaal crust from the Vredefort Dome, Johannesburg Dome and Southern Marginal Zone (SMZ) of the Archean Limpopo Belt. Composition of the middle crust used in this simulation (shown as pentagram) is situated at the transition region between the regional TTG gneisses/granites and pelitic-greywacke gneisses, and is also similar to the estimated composition of middle crusts from Weaver & Tarney[50] and Shaw et al.[51]. All data are collected from multiple sources cited in the supplementary references[52-72]. Source Data are provided as a Source Data file.

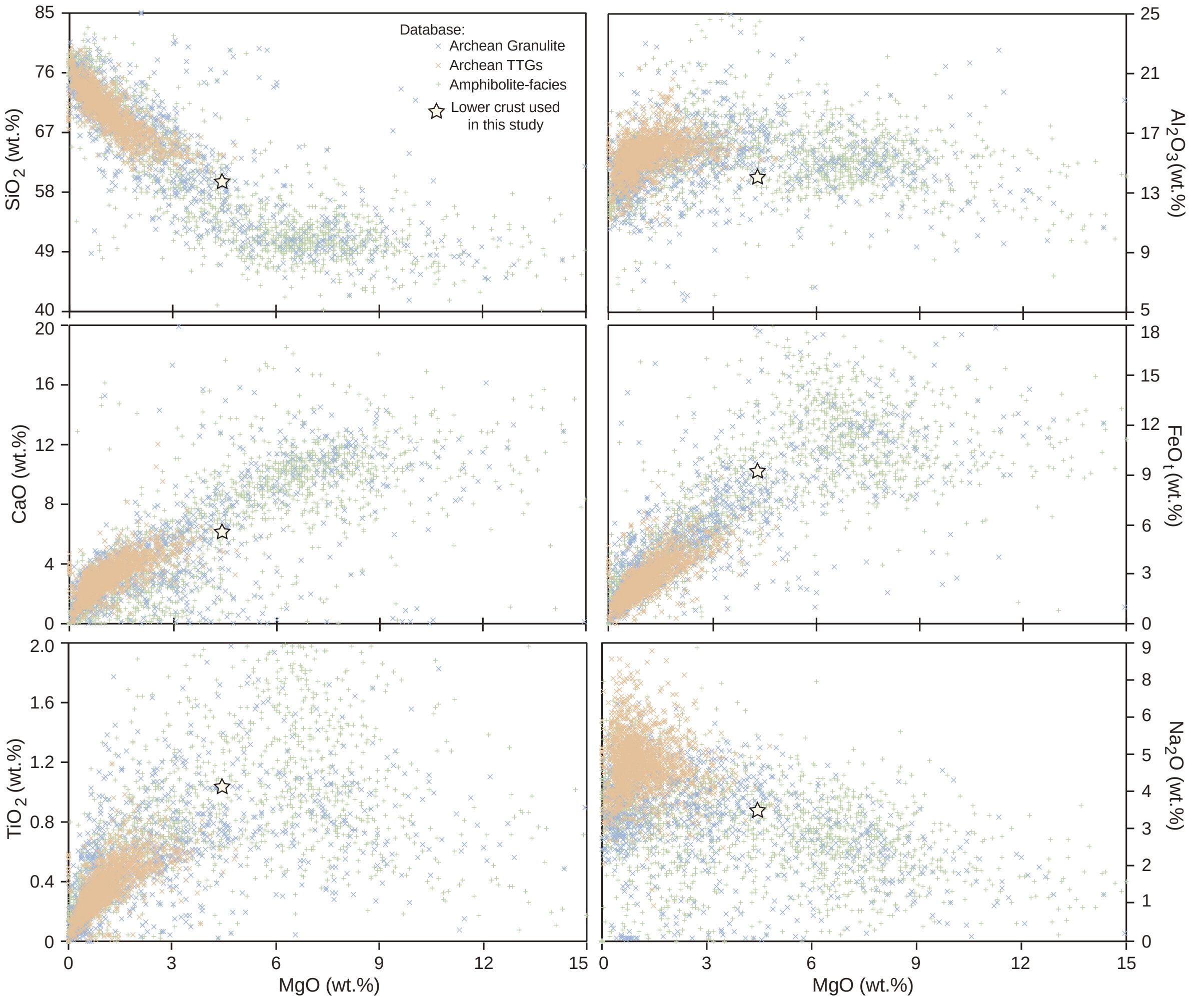

Supplementary Figure 5. MgO against **a-e** SiO_2, Al_2O_3, CaO, FeO_t, Na_2O and K_2O for the worldwide database of Archean rock compositions of deeply exhumed, lower-crustal, granulite-facies and tonalite-trondhjemite-granodiorites (TTGs) terrains, as well as the database of lower-crustal, amphibolite-facies samples. Composition of the lower crust used in this simulation comes from the estimated composition of granulite terrain from the interior of the North China Craton[20], and is shown as pentagram. All data are collected from multiple sources cited in the supplementary references[73-75]. Source data are provided as a Source Data file.

Supplementary Table 1. Modeling parameters for the Scenario 1 in integration stage of RLS.

	Dun (LZ)	Hz (LZ)	Px (LZ)	Px (CZ$_L$)	B1
Mode	Assimilate upper crust at 2 kbar and 300 ºC				
Assimilation degree (%)	17.36	22.48	27.01	33.77	33.77
Temperature after assimilation (ºC)	1379	1335	1293	1222	1222
Solid proportions (%)	13.1	21.6	28.5	37.3	37.3
Solid: liquid ratio in mixture	95.5:4.5	82.2:17.8	85.5:14.5	85:15	78:22

Supplementary Table 2. Modeling parameters for the Scenario 2 in integration stage of RLS.

		No (CZ$_U$)	B2	Gn (MZ)	B3
Assimilation + cooling in deep magma reservoirs	Mode	Assimilate middle crust as 4.5 kbar and 390 ºC			
	Assimilation degree (%)	20.62		24.23	
	Temperature (ºC) before ascent	1237		1255	
	Solid: liquid ratio before ascent	49.9:50.1		44.7:55.3	
	Detained solid (%)[a]	90%		97%	
Ascent + cooling +emplacement	Temperature (ºC)	1181	1181	1130	1181
	Crystallization degree (%)	40.56	40.56	63.92	34.18
	Solid: liquid ratio In mixture	95:5	5:95	97:3	42:58

[a] Relative to the whole solid phases after bulk assimilation and cooling.

Supplementary Table 3. Compositions of primitive komatiite and crustal assimilants.

Element	Komatiite	UC	MC	LC	UUMZ[a]	Element	Komatiite	UC	MC
SiO_2	47.61	69.45	69.09	59.66	51.85	Tb	0.16	0.7	0.34
TiO_2	0.37	0.64	0.22	1.04	1.38	Dy	1.17	3.9	1.7
Al_2O_3	9.64	12.98	16.07	13.97	15.36	Ho	0.26	0.83	0.33
Cr_2O_3	0.49	0.0136	0.10	0.018	0.04	Er	0.83	2.3	0.85
FeO	10.86	5.536	3.69	9.28	12.49	Tm	0.12	0.3	0.2
MnO	0.20	0.10	0.05	0.16	0.4	Yb	0.84	1.96	1.37
MgO	22.61	2.01	1.40	4.45	3.94	Lu	0.13	0.31	0.31
CaO	8.08	3.32	4.01	6.19	8.88	Rb	0.53	84	46.44
Na_2O	0.12	3.12	4.16	3.49	2.78	Ba	7.45	628	446.62
K_2O	0.03	2.82	1.30	1.75	1.67	Th	0.1	10.5	3.86
H_2O	0	1.0	0.5	0.5	0.48	U	0.03	2.7	0.6
La	0.64	31	31.03	-	-	Nb	0.79	12	11.13
Ce	1.72	63	47.15	-	-	Ta	0.05	0.9	0.68
Pr	0.26	7.1	5.37	-	-	Sr	28	320	492.91
Nd	1.29	27	18.16	-	-	Zr	17.03	193	113.6
Sm	0.55	4.7	3.03	-	-	Hf	0.57	5.3	3.01
Gd	0.84	4	2.33	-	-	Y	8.51	21	12.92

[a] Modeled composition of the incoming parental magma for the *UUMZ*.

Supplementary Table 4. Isotopic compositions of potential crustal assimilants.

		$\delta^{18}O$	$(^{87}Sr/^{86}Sr)_{2050}$	ε_{Nd} at 2050 Ma
Pretoria Group	Dullstroom Fm.	5.96±0.74 (3)	-	-
	Quartzite	10.65±1.09 (14)	-	-5.8±1.8 (9)
	Metapelite	12.4±0.98 (24)	-	
	Dolomite[a]	20.49±2.66 (7)	0.7038	-6.84
Rooiberg Group		7.36±1.86 (4)	0.7068±0.002 (43)	-7.8±1.3 (59)
Rashoop Granophyre Suite		6.6±1.0 (15)	-	-5.3±3.2 (20)
Lebowa Granite Suite		7.35±1.04 (40)	-	
**Upper Crust**		**9.6**[b]	**0.706**[b]	**-6.8**[b]
Vredefort Dome	Metapelite	8.15±2.92 (27)	0.7105±0.0086[c] (65)	-13.51±4.2[c] (26)
	Gneiss	9.39±1.27 (52)		
SMZ, Limpopo Belt	Metapelite	9.9±1.13 (53)	0.7059±0.0032 (40)	-7.09±1.4 (19)
	Gneiss	7.72±0.51 (28)	0.7089±0.0065 (94)	-8.61±2.7 (49)
**Middle Crust**		**10.6**[b]	**0.7088**±0.0069 (199)	**-9.66**±3.8 (94)
**Lower Crust**		**9.2**±2.2 (28)	**0.7080**±0.0068 (21)	**-7.45**±5.1 (17)

Numbers in brackets are the samples collected. All data are collected from multiple sources cited in the supplementary references[13,19,32,34,36,56,61,63,76-114]. Most isotopic compositions of the contaminants are assumed as the average values of collected data, and the corresponding standard deviations are shown as the numbers behind the sign of "±".

[a] Direct contact with the RLS at Sandsloot Mine[105], Northern Limb.

[b] Assumed values in the assimilation modelling.

[c] Samples from the Johannesburg Dome are included, except the granites with extremely high $(^{87}Sr/^{86}Sr)_{2050}$ values, 0.7675±0.0182 (22).

Supplementary Table 5. Operating process for alphaMELTS modeling

****

Operational process for the ABC and AFC via AlphaMELTS

****

1	% After opening the AlphaMELTS, option 1 to read the MELTS file to set the composition of parental melt
/../../../Komatiite.melts	% type the file path of parental melt "Komatiite.melt", and then AlphaMELTS read the compositions of parental melt
2	% set or modify starting temperature (C) and pressure (bars) of the parental melt. Enter a value <=0.0 to retain the old temperature and/or pressure
3	% Single calculation for the equilibrium state at the starting P-T conditions
1	% Calculation use the superliquidus as initial guess, the output gives the liquid temperature of parental magma under the pressure setting
4	% Do the assimilation calculations
100	% Set the number of increment assimilation calculation, if the temperature of system drop to the value below the minimum temperature in the setting file, the calculation will cease although the calculation time may not reach this setting number here
0 or negative value	% Here, there is two way to set the compositions of assimilant:
	% "0" for binary input file. The binary restart file from AlphaMELTS is used to set the composition of assimilant. We suggest this way because the binary restart file contain many thermodynamic informations of the assimilant, e.g., enthalpy, which can improve the accuracy of assimilation calculation. But, you can not directly input the MELTS file of assimilant (e.g., Upper_crust.melts, Middle_crust.melts, Lower_crust.melts). AlphaMELTS should firstly do a single calculation with the superliquidus start, for the composition, pressure and temperature in the assimilant MELST file, and then restore the whole system into a binary restart file.
	% "negative value" for separate MELTS files for each phase. Many separate files are used to represent the compositions and modes of different minerals in the assimilant. Then you should use the MELTS phase name and then the file name e.g., 'feldspar', 'feldspar.melts', 'quartz', 'quartz.melts'.... to input these files in the assimilation calculations. Here, the melts files for each mineral phase should contain their respective mass mode in the assimilant.
1	% enter the mass of assimilant to be the added in grams per cycle for the assimilation
/../../Upper_crust	% type the path of the binary restart file or the MELTS phase name and the separate MELTS files for each phase in assimilant
x	% terminate the assimilation calculations
0	% Exits

Supplementary Table 6. alphaMELTS environment file for ABC

```
! ************************************
!  Assimilation-batch crystallization (ABC) of crust
! ************************************
! this variable chooses MELTS or pMELTS; for low-pressure use MELTS
ALPHAMELTS_VERSION        MELTS
ALPHAMELTS_CELSIUS_OUTPUT   true
! don't use this unless fO2 anomalies at the solidus are a problem
! ALPHAMELTS_ALTERNATIVE_FO2    true
! use this if you want to buffer fO2 for isentropic, isenthalpic or isochoric mode
! e.g. if you are doing isenthalpic AFC
! ALPHAMELTS_IMPOSE_FO2 true
! use if you want assimilation and fractional crystallization (AFC)
ALPHAMELTS_ASSIMILATE   true
! isothermal, isobaric, isentropic, isenthalpic, isochoric, geothermal or PTPath
ALPHAMELTS_MODE         isenthalpic
!ALPHAMELTS_PTPATH_FILE    ptpath.txt
! need to set DELTAP for polybaric paths; DELTAT for isobaric paths
ALPHAMELTS_DELTAP       0
ALPHAMELTS_DELTAT       0
ALPHAMELTS_MAXP         30000
ALPHAMELTS_MINP         1
ALPHAMELTS_MAXT         2000
ALPHAMELTS_MINT         900
! this one turns on fractional crystallization for all solids
! use 'Fractionate:' in the melts file instead for selective fractionation
!ALPHAMELTS_FRACTIONATE_SOLIDS          true
!ALPHAMELTS_MASSIN       0.001
! free water is unlikely but can be extracted
!ALPHAMELTS_FRACTIONATE_WATER      true
!ALPHAMELTS_MINW         0.9
! the next six options refer to the trace element engine
ALPHAMELTS_DO_TRACE           true
!ALPHAMELTS_DO_TRACE_H2O      true
!ALPHAMELTS_HK_OL_TRACE_H2O       true
!ALPHAMELTS_HK_PXGT_TRACE_H2O     mineral-mineral
!ALPHAMELTS_2X_OPX_TRACE_H2O     true
ALPHAMELTS_TRACE_DEFAULT_DPTX true
!ALPHAMELTS_TRACE_NORMALIZATION 1
!ALPHAMELTS_TRACE_INPUT_FILE   yourtraceinfile.txt
ALPHAMELTS_TRACE_USELIQFEMG   true
! the next one gives an output file that is always updated, even for single calculations
!ALPHAMELTS_SAVE_ALL true
!ALPHAMELTS_SKIP_FAILURE true
! this information overwrites stuff in the initial melts file
! a new melts file is created if this information is different
!Initial Temperature: 1400
!Initial Pressure: 500
!Log fO2 Path: FMQ
```

Supplementary Table 7. alphaMELTS environment file for AFC.

```
! ***************************************
!  Assimilation-fractional crystallization (AFC) of crust
! ***************************************
! this variable chooses MELTS or pMELTS; for low-pressure use MELTS
ALPHAMELTS_VERSION        MELTS
ALPHAMELTS_CELSIUS_OUTPUT   true
! don't use this unless fO2 anomalies at the solidus are a problem
! ALPHAMELTS_ALTERNATIVE_FO2    true
! use this if you want to buffer fO2 for isentropic, isenthalpic or isochoric mode
! e.g. if you are doing isenthalpic AFC
! ALPHAMELTS_IMPOSE_FO2 true
! use if you want assimilation and fractional crystallization (AFC)
ALPHAMELTS_ASSIMILATE   true
! isothermal, isobaric, isentropic, isenthalpic, isochoric, geothermal or PTPath
ALPHAMELTS_MODE         isenthalpic
!ALPHAMELTS_PTPATH_FILE    ptpath.txt
! need to set DELTAP for polybaric paths; DELTAT for isobaric paths
ALPHAMELTS_DELTAP       0
ALPHAMELTS_DELTAT       0
ALPHAMELTS_MAXP         30000
ALPHAMELTS_MINP         1
ALPHAMELTS_MAXT         2000
ALPHAMELTS_MINT         900
! this one turns on fractional crystallization for all solids
! use 'Fractionate:' in the melts file instead for selective fractionation
ALPHAMELTS_FRACTIONATE_SOLIDS           true
ALPHAMELTS_MASSIN       0.001
! free water is unlikely but can be extracted
!ALPHAMELTS_FRACTIONATE_WATER      true
!ALPHAMELTS_MINW         0.9
! the next six options refer to the trace element engine
ALPHAMELTS_DO_TRACE           true
!ALPHAMELTS_DO_TRACE_H2O      true
!ALPHAMELTS_HK_OL_TRACE_H2O       true
!ALPHAMELTS_HK_PXGT_TRACE_H2O     mineral-mineral
!ALPHAMELTS_2X_OPX_TRACE_H2O     true
ALPHAMELTS_TRACE_DEFAULT_DPTX true
!ALPHAMELTS_TRACE_NORMALIZATION 1
!ALPHAMELTS_TRACE_INPUT_FILE   yourtraceinfile.txt
ALPHAMELTS_TRACE_USELIQFEMG   true
! the next one gives an output file that is always updated, even for single calculations
!ALPHAMELTS_SAVE_ALL true
!ALPHAMELTS_SKIP_FAILURE true
! this information overwrites stuff in the initial melts file
! a new melts file is created if this information is different
!Initial Temperature: 1400
!Initial Pressure: 500
!Log fO2 Path: FMQ
```

Supplementary Table 8. alphaMELTS .melts file for komatiite.
Title: NMXD43-3
Initial Composition: SiO2 47.5402
Initial Composition: TiO2 0.3706
Initial Composition: Al2O3 9.6262
Initial Composition: Fe2O3 1.4267
Initial Composition: Cr2O3 0.4846
Initial Composition: FeO 9.5592
Initial Composition: MnO 0.2003
Initial Composition: MgO 22.5781
Initial Composition: CaO 8.0636
Initial Composition: Na2O 0.1202
Initial Composition: K2O 0.0301
Initial Composition: P2O5 0.00
Initial Composition: H2O 0.00
Initial Temperature: 1547.07
Initial Pressure: 2000.00
Initial Trace: La 0.64
Initial Trace: Ce 1.72
Initial Trace: Pr 0.26
Initial Trace: Nd 1.29
Initial Trace: Sm 0.55
Initial Trace: Eu 0.16
Initial Trace: Gd 0.84
Initial Trace: Tb 0.16
Initial Trace: Dy 1.17
Initial Trace: Ho 0.26
Initial Trace: Er 0.83
Initial Trace: Tm 0.12
Initial Trace: Yb 0.84
Initial Trace: Lu 0.13
Initial Trace: Rb 0.53
Initial Trace: Ba 7.45
Initial Trace: Th 0.1
Initial Trace: U 0.03
Initial Trace: Nb 0.79
Initial Trace: Ta 0.05
Initial Trace: P 130.9262
Initial Trace: Sr 28
Initial Trace: Zr 17.03
Initial Trace: Hf 0.57
Initial Trace: Y 8.51
Initial Trace: Pb 0.93

Supplementary Table 9. alphaMELTS .melts file for upper crust.
Title: Upper crust from the Supplementary Figure 3
Initial Composition: SiO2 69.0
Initial Composition: TiO2 0.64
Initial Composition: Al2O3 12.9
Initial Composition: Fe2O3 0.064
Initial Composition: Cr2O3 0.0135
Initial Composition: FeO 5.44
Initial Composition: MnO 0.1
Initial Composition: MgO 2.00
Initial Composition: CaO 3.3
Initial Composition: Na2O 3.1
Initial Composition: K2O 2.8
Initial Composition: P2O5 0.00
Initial Composition: H2O 1.00
Initial Temperature: 300
Initial Pressure: 2000.00
Increment Temperature: 0.00
Increment Pressure: 0.00
log fo2 Path: QFM
Log fO2 Delta: 0.0
Suppress: olivine
Suppress: garnet
Suppress: orthopyroxene
Initial Trace: La 31
Initial Trace: Ce 63
Initial Trace: Pr 7.1
Initial Trace: Nd 27.0
Initial Trace: Sm 4.7
Initial Trace: Eu 1.0
Initial Trace: Gd 4.0
Initial Trace: Tb 0.7
Initial Trace: Dy 3.9
Initial Trace: Ho 0.83
Initial Trace: Er 2.3
Initial Trace: Tm 0.3
Initial Trace: Yb 1.96
Initial Trace: Lu 0.31
Initial Trace: Rb 84
Initial Trace: Ba 628
Initial Trace: Th 10.5
Initial Trace: U 2.7
Initial Trace: Nb 12
Initial Trace: Ta 0.9
Initial Trace: P 654.63
Initial Trace: Sr 320
Initial Trace: Zr 193
Initial Trace: Hf 5.3
Initial Trace: Y 21
Initial Trace: Pb 17

Supplementary Table 10. alphaMELTS .melts file for middle crust.
Title: Middle crust from Supplementary Figure 4
Initial Composition: SiO2 69.093046
Initial Composition: TiO2 0.2156024
Initial Composition: Al2O3 16.074913
Initial Composition: Fe2O3 0.0250588
Initial Composition: Cr2O3 0.10
Initial Composition: FeO 3.64127
Initial Composition: MnO 0.0501401
Initial Composition: MgO 1.4039226
Initial Composition: CaO 4.0112073
Initial Composition: Na2O 4.1616276
Initial Composition: K2O 1.3036424
Initial Composition: P2O5 0.00
Initial Composition: H2O 0.50
Initial Temperature: 390.00
Initial Pressure: 4500.00
Increment Temperature: 0.00
Increment Pressure: 0.00
log fo2 Path: QFM
Log fO2 Delta: 0.0
Suppress: olivine
Suppress: garnet
Suppress: orthopyroxene
Limit coexisting: clinopyroxene 0
Initial Trace: La 31.03
Initial Trace: Ce 47.15
Initial Trace: Pr 5.37251
Initial Trace: Nd 18.16
Initial Trace: Sm 3.03
Initial Trace: Eu 0.84
Initial Trace: Gd 2.33
Initial Trace: Tb 0.3401244
Initial Trace: Dy 1.7
Initial Trace: Ho 0.33242186
Initial Trace: Er 0.85
Initial Trace: Tm 0.20374762
Initial Trace: Yb 1.37
Initial Trace: Lu 0.31
Initial Trace: Rb 46.44
Initial Trace: Ba 446.62
Initial Trace: Th 3.86
Initial Trace: U 0.6
Initial Trace: Nb 11.13
Initial Trace: Ta 0.68
Initial Trace: P 654.63
Initial Trace: Sr 492.91
Initial Trace: Zr 113.6
Initial Trace: Hf 3.01
Initial Trace: Y 12.9171429

Initial Trace: Pb 13.3

Supplementary Table 11. alphaMELTS .melts file for lower crust.
Title: Lower crust
Initial Composition: SiO2 59.66
Initial Composition: TiO2 1.04
Initial Composition: Al2O3 13.97
Initial Composition: Fe2O3 0.049
Initial Composition: Cr2O3 0.01797714
Initial Composition: FeO 9.3
Initial Composition: MnO 0.16
Initial Composition: MgO 4.45
Initial Composition: CaO 6.20
Initial Composition: Na2O 3.5
Initial Composition: K2O 1.75
Initial Composition: P2O5 0.00
Initial Composition: H2O 0.50
Initial Temperature: 770.00
Initial Pressure: 10000.00
Increment Temperature: 0.00
Increment Pressure: 0.00
log fo2 Path: QFM
Log fO2 Delta: -2.0
Suppress: olivine
Limit coexisting: clinopyroxene 2
Initial Trace: La 29
Initial Trace: Ce 53
Initial Trace: Pr 2.4
Initial Trace: Nd 25
Initial Trace: Sm 4.65
Initial Trace: Eu 1.39
Initial Trace: Gd 3.1
Initial Trace: Tb 0.86
Initial Trace: Dy 3.1
Initial Trace: Ho 0.68
Initial Trace: Er 1.9
Initial Trace: Tm 0.24
Initial Trace: Yb 2.29
Initial Trace: Lu 0.38
Initial Trace: Rb 56
Initial Trace: Ba 509
Initial Trace: Th 5.23
Initial Trace: U 0.86
Initial Trace: Nb 10
Initial Trace: Ta 0.6
Initial Trace: P 916.483403
Initial Trace: Sr 308
Initial Trace: Zr 162
Initial Trace: Hf 4.2
Initial Trace: Y 18

Supplementary References

1. Wilson, A. H. A chill sequence to the Bushveld Complex: Insight into the first stage of emplacement and implications for the parental magmas. *J. Petrol.* **53**, 1123-1168 (2012).
2. Wilson, A. H. The earliest stages of emplacement of the Eastern Bushveld Complex: Development of the Lower Zone, Marginal Zone and Basal Ultramafic Sequence. *J. Petrol.* **56**, 347-388 (2015).
3. Wilson, A. & Chunnett, G. Trace element and platinum group element distributions and the genesis of the Merensky Reef, Western Bushveld Complex. *J. Petrol.* **47**, 2369-2403 (2006).
4. Yudovskaya, M. A., Kinnaird, J. A., Sobolev, A. V., Kuzmin, D. V., McDonald, I. & Wilson, A. H. Petrogenesis of the Lower Zone olivine-rich cumulates beneath the Platreef and their correlation with recognized occurrences in the Bushveld Complex. *Econ. Geol.* **108**, 1923-1952 (2013).
5. Teigler, B. Mineralogy, petrology and geochemistry of the Lower and Lower Critical Zone, northwestern Bushveld Complex. Ph.D. thesis, Rhodes University, Grahamstown (1990).
6. Mungall, J. E., Kamo, S. L. & McQuade, S. U-Pb geochronology documents out-of-sequence emplacement of ultramafic layers in the Bushveld Igneous Complex of South Africa. *Nat. Commun.* **7**, 13385 (2016).
7. Arndt, N., Jenner, G., Ohnenstetter, M., Deloule, E. & Wilson, A.H. Trace elements in the Merensky Reef and adjacent norites Bushveld Complex South Africa. *Miner. Deposita* **40**, 550-575 (2005).
8. Cawthorn, R. G. & Barry, S. D. The role of intercumulus residua in the formation of pegmatoid associated with the UG2 chromitite, Bushveld Complex. *Aust J. Earth Sci.* **39**, 263-276 (1992).
9. Barnes, S. J. & Maier, W. D. Platinum-group elements and microstructures of normal Merensky Reef from Impala Platinum Mines, Bushveld Complex. *J. Petrol.* **43**, 103-128 (2002).
10. Hardwick, B. W., Jowitt, S. M. & Keays, R. R. Controls on disseminated PGE-Cu-Ni sulfide mineralization within the Rietfontein deposit, Eastern Limb, Bushveld Complex, South Africa: Implications for the formation of contact-type magmatic sulfide deposits. *Ore Geol. Rev.* **64**, 253-272 (2015).
11. Hutchinson, D., Foster, J., Prichard, H. & Gilbert, S. Concentration of particulate Platinum-Group minerals during magma emplacement: a case study from the Merensky Reef,

Bushveld Complex. *J. Petrol.* **56**, 113-159 (2015).

12. Ihlenfeld, C. & Keays, R. R. Crustal contamination and PGE mineralization in the Platreef, Bushveld Complex, South Africa: evidence from multiple contamination events and transport of magmatic sulfides. *Miner. Deposita* **46**, 813-832 (2011).
13. Lundgaard, K. L., Tegner, C., Cawthorn, R. G., Kruger, F. J. & Wilson, J. R. Trapped intercumulus liquid in the Main Zone of the eastern Bushveld Complex, South Africa. *Contrib. Mineral. Petrol.* **151**, 352-369 (2006).
14. Maier, W. D. Geochemical and petrological trends in the UG2-Merensky Unit interval of the Upper Critical Zone in the Western Bushveld Complex. Ph.D., thesis, Rhodes University, Grahamstown (1992).
15. Maier, W. D., Barnes, S. J. & Groves, D. I. The Bushveld Complex, South Africa: formation of platinum-palladium, chrome- and vanadium-rich layers via hydrodynamic sorting of a mobilized cumulate slurry in a large, relatively slowly cooling, subsiding magma chamber. *Miner. Deposita* **48**, 1-56 (2013).
16. Maier, W. D., Barnes, S. J. & Karykowski, B. T. A chilled margin of komatiite and Mg-rich basaltic andesite in the western Bushveld Complex, South Africa. *Contrib. Mineral. Petrol.* **171**, 57 (2016).
17. Maier, W. D., de Klerk, L., Blaine, J., Manyeruke, T., Barnes, S.J., Stevens, M. V. A. & Mavrogenes, J.A. Petrogenesis of contact-style PGE mineralization in the northern lobe of the Bushveld Complex: comparison of data from the farms Rooipoort, Townlands, Drenthe and Nonnenwerth. *Miner. Deposita* **43**, 255-280 (2008).
18. Mitchell, A. A. The petrology, mineralogy and geochemistry of the Main Zone of the Bushveld Complex at Rustenburg Platinum Mine, Union Section. Ph.D. thesis, Rhodes University, Grahamstown, p. 157 (1986).
19. Roelofse, F. & Ashwal, L. D. The Lower Main Zone in the Northern Limb of the Bushveld Complex- a > 1.3 km thick sequence of intruded and variably contaminated crystal mushes. *J. Petrol.* **53**, 1449-1476 (2012).
20. Gao, S., Luo, T. C., Zhang, B. R., Zhang, H. F., Han, Y. W., Zhao, Z. D. & Hu, Y. K. Chemical composition of the continental crust as revealed by studies in East China. *Geochim. Cosmochim. Acta* **62**, 1959-1975 (1998).
21. Rudnick, R. L. & Gao, S. Composition of the continental crust. In, Turekian, K. & Holland, H. (eds). Treatise on Geochemistry, 2nd Edition, Elsevier Science, Vol. 4, 1-51 (2014).
22. Buchanan, P. C., Koeberl, C. & Reimold, W. U. Petrogenesis of the Dullstroom Formation, Bushveld Magmatic Province, South Africa. *Contrib. Mineral. Petrol.* **137**, 133-146

(1999).

23. Buchanan, P. C., Reimold, W. U., Koeberl, C. & Kruger, F. J. Geochemistry of intermediate to silicate volcanic rocks of the Rooiberg Group, Bushveld Magmatic Province, South Africa. *Contrib. Mineral. Petrol.* **144**, 131-143 (2002).
24. Button, A. Low-potash pillow basalts in the Pretoria Group, Transvaal Supergroup. *S. Afr. J. Geol.* **77**, 99-104 (1977).
25. Coetzee, L. L., Beukes, N. J., Gutzmer, J. & Kakegawa, T. Links of organic carbon cycling and burial to depositional depth gradients and establishment of a snowball Earth at 2.3 Ga. Evidence from the Timeball Hill Formation, Transvaal Supergroup, South *Africa. S. Afr. J. Geol.* **109**, 109-122 (2006).
26. Crow, C. & Condie, K. C. Geochemistry and origin of early Proterozoic volcanic rocks from the Transvaal and Soutpansberg successions, South Africa. *Precambrian Res.* **47**, 17-26 (1990).
27. Eriksson, P. G., Twist, D., Snyman, C. P. & Burger, L. The geochemistry of the Silverton Shale Formation, Transvaal Sequence. *S. Afr. J. Geol.* **93**, 454-462 (1990).
28. Eriksson, P. G., Engelbrecht, J. P., Res, M. & Harmer, R. E. The Bushy Bend lavas, a new volcanic member of the Pretoria Group, Transvaal Sequence. *S. Afr. J. Geol.* **97**, 1-7 (1994).
29. Eriksson, P. G., Reczko, B. F. F., Merkle, R. K. W., Schreiber, U. M., Engelbrecht, J. P., Res, M. & Snyman, C. P. Early Proterozoic black shales of the Timeball Hill Formation, South Africa: volcanogenic and palaeoenvironmental influences. *J. Afr. Earth Sci.* **18**, 325-337 (1994).
30. Eriksson, P. G., Schweitzer, J. K., Bosch, P. J. A., Schereiber, U. M., van Deventer, J. L. & Hatton, C. J. The Transvaal Sequence: an overview. *J. Afr. Earth Sci.* **16**, 25-51 (1993).
31. Eriksson, P. G. Schreiber, U. M., Reczko, B. F. E. & Snyman, C. P. Petrography and geochemistry of sandstones interbedded with the Rooiberg Felsite Group (Transvaal Sequence, South Africa): implications for provenance and tectonic setting. *J. Sediment. Res.* **64**, 836-846 (1994).
32. Günther, T., Haase, K. M., Klemd, R. & Teschner, C. Mantle sources and magma evolution of the Rooiberg lavas, Bushveld Large Igneous Province, South Africa. *Contrib. Mineral. Petrol.* **173**, 51 (2018).
33. Hatton, C. J. & Schweitzer, J. K. Evidence for synchronous extrusive and intrusive Bushveld magmatism. *J. Afr. Earth Sci.* **21**, 579-594 (1995).
34. Hill, M., Barker, F., Hunter, D. & Knight, R. Geochemical characteristics and origin of the

Lebowa Granite Suite, Bushveld Complex. *Int. Geol. Rev.* **38**, 195-227 (1996).

35. Humbert, F., de Kock, M. O., Altermann, W., Elburg, M. A., Lenhardt, N., Smith, A. J. B. & Masango, S. Petrology, physical volcanology and geochemistry of a Paleoproterozoic large igneous province: The Hekpoort Formation in the southern Transvaal sub-basin (Kaapvaal craton). *Precambrian Res.* **315**, 232-256 (2018).
36. Jahn, B. M. & Condie, K. C. Evolution of the Kaapvaal Craton as viewed from geochemical and Sm-Nd isotopic analyses of intracratonic pelites. *Geochim. Cosmochim. Acta* **59**, 2239-2258 (1995).
37. Jolayemi, O. O. Chemical evolution of the Paleoproterozoic Rooiberg Group, Kaapvaal Craton, South Africa: new insights into the formation of a silicic large igneous province (SLIP). Ph. D. thesis, University of Pretoria, p. 133 (2015).
38. Oberholzer, J. D. & Eriksson, P. G. Subaerial volcanism in the Palaeoproterozoci Hekpoort Formation (Transvaal Supergropu), Kaapvaal craton. *Precambrian Res.* **101**, 193-201 (2000).
39. Schreiber, U. M. A Palaeoenvironmental study of the Pretoria Group in the eastern Transvaal. Ph. D. thesis, University of Pretoria, Pretoria, p. 308 (1990).
40. Schreiber, U. M., Eriksson, P. G. & Snyman, C. P. A provenance study of the sandstones of the Pretoria Group, Transvaal Sequence (South Africa): petrography, geochemistry, and palaeocurrent directions. *S. Afr. J. Geol.* **94**, 288-298 (1991).
41. Schreiber, U. M., Eriksson, P. G. & Snyman, C. P. Mudrock geochemistry of the Proterozoic Pretoria Group, Transvaal Sequence (South Africa): geological implications. *J. Afr. Earth Sci.* **14**, 393-409 (1992).
42. Schreiber, U. M., Eriksson, P. G., van der Neut, M. & Snyman, C. P. Sedimentary petrography of the Early Proterozoic Pretoria Group, Transvaal Sequence, South Africa: implications for tectonic setting. *Sediment. Geol.* **80**, 89-103 (1992).
43. Schweitzer, J. K. The Dullstroom Basalt Formation and the Rooiberg Group: volcanic rocks associated with the Bushveld Complex. Dissertation, University of Pretoria (1998).
44. Schweitzer, J. K. & Hatton, C. J. Chemical alteration within the volcanic roof rocks of the Bushveld Complex. *Econ. Geol.* **90**, 2218-2231 (1995).
45. Schweitzer, J. K., Hatton, C. J. & de Waal, S. A. Link between the granitic and volcanic rocks of the Bushveld Complex, South Africa. *J. Afr. Earth Sci.* **24**, 95-104 (1997).
46. Twist, D. Geochemical evolution of the Rooiberg Silicic lavas in the Loskop Dam Area, Southeastern Bushveld. *Econ. Geol.* **80**, 1153-1165 (1985).
47. Twist, D. & Harmer, R. E. J. Geochemistry of contrasting siliceous magmatic suites in the

Bushveld Complex: genetic aspects and implications for tectonic discrimination diagrams. *J. Volcanol. Geotherm. Res.* **32**, 83-98 (1987).

48. Walraven, F. Textrual, geochemical and genetic aspects of the granophyric rocks of the Bushveld Complex. Memoris, Geological Survey of South Africa 72, p. 145 (1987).
49. Wronkiewicz, D. J. & Condie, K. C. Geochemistry and mineralogy of sediments from the Ventersdorp and Transvaal Supergroups, South Africa: cratonic evolution during the early Proterozoic. *Geochim. Cosmochim. Acta* **54**, 343-354 (1990).
50. Shaw, D. M., Dickin, A. P., Li, H., McNutt, R. H., Schwarcz, H. P. & Truscott, M. G. Crustal geochemistry in the Wawa-Foleyet region, Ontario. *Can. J. Earth Sci.* **31**, 1104-1121 (1994).
51. Weaver, B. L. & Tarney, J. Empirical approach to estimating the composition of the continental crust. *Nature* **310**, 575-577 (1984).
52. Anhaeusser, C. R. Structures in granitoid gneisses and associated migmatites close to the granulite boundary of the Limpopo Belt, South Africa. *Precambrian Res.* **55**, 81-92 (1992).
53. Bohlender, F., van Reenen, D. D. & Barton, J. M. Evidence for metamorphic and igneous charnockites in the Southern Marginal Zone of the Limpopo Belt. *Precambrian Res.* **55**, 429-449 (1992).
54. Chavagnac, V. Behaviour of the Sm-Nd isotopic system during metamorphism: Examples from the HT-LP metamorphic terrane of the Limpopo Belt, Sourth Africa and the UHP metamorphic terrane of Dabeishan, Central China. Applied Geology. Université Rennes 1 (1999).
55. Henderson, D. R., Long, L. E. & Barton, J. M. Isotopic ages and chemical and isotopic composition of the Archaean Turfloop Batholith, Pietersburg granite-greenstone terrane, Kaapvaal Craton, South Africa. *S. Afr. J. Geol.* **103**, 38-46 (2000).
56. Kreissig, K., Nägler, T. F., Kramers, J. D., van Reenen, D. D. & André Smit, C. An isotopic and geochemical study of the northern Kaapvaal Craton and the Southern Marginal Zone of the Limpopo Belt: are they juxtaposed terranes? *Lithos* **50**, 1-25 (2000).
57. Nicoli, G., Stevens, G., Moyen, J. F., Vezinet, A. & Mayne, M. Insights into the complexity of crustal differentiation: K2O-poor leucosomes within metasedimentary migmatites from the Southern Marginal Zone of the Limpopo Belt, South Africa. *J. Metamorph. Geol.* **35**, 999-1022 (2017).
58. Nicoli, G., Stevens, G., Moyen, J. F. & Frei, D. Rapid evolution from sediment to anatectic granulite in an Archean continental collision zone: the example of the Bandelierkop

Formation metapelites, South Marginal Zone, Limpopo Belt, South Africa. *J. Metamorph. Geol.* **33**, 177-202 (2015).

59. Smit, C. A. & van Reenen, D. D. Deep crustal shear zones, high-grade tectonites, and associated metasomatic alteration in the Limpopo Belt, South Africa: Implications for deep crustal processes. *J. Geol.* **105**, 37-57 (1997).
60. Taylor, J., Nicoli, G., Stevens, G., Frei, D. & Moyen, J. F. The processes that control leucosome compositions in metasedimentary granulites: perspectives from the Southern Marginal Zone migmatites, Limpopo Belt, South Africa. *J. Metamorph. Geol.* **32**, 713-742 (2014).
61. Hart, R. J., Anderoli, M. A. G., Tredoux, M. & De Wit, M. J. Geochemistry across an exposed section of Archaean crust at Vredefort, South Africa: with implications for mid-crustal discontinuities. *Chem. Geol.* **82**, 21-50 (1990).
62. Hart, R. J., McDonald, I., Tredoux, M., de Wit, M. J., Carlson, R. W., Andreoli, M., Moser, D. E. & Ashwal, L. D. New PGE and Re/Os-isotope data from lower crustal sections of the Vredefor Dome and a reinterpretation of its "crust on edge" profile. *S. Afr. J. Geol.* **107**, 173-184 (2004).
63. Hart, R. J., Welke, H. J. & Nicolaysen, L. O. Geochronology of the deep profile through Archean Basement at Vredefort, with implications for early crust evolution. *J Geophys. Res.* **86**, 10663-10680 (1981).
64. Lieger, D., Riller, U. & Gibson, R. L. Petrographic and geochemical evidence for an allochthonous, possibly impact melt, origin of pseudotachylite from the Vredefort Dome, South Africa. *Geochim. Cosmochim. Acta* **75**, 4490-4514 (2011).
65. Lana, C., Reimold, W. U., Gibson, R. L., Koeberl, C. & Siegesmund, S. Nature of the Archean midcrust in the core of the Vredefort Dome, Central Kaapvaal Craton, South Africa. *Geochim. Cosmochim. Acta* **68**, 623-642 (2004).
66. Ogilvie, P. Metamorphic studies in the Vredefort dome, South Africa. Thesis, University of the Witwatersrand, p. 737 (2010).
67. Reimold, W. U., Pybus, G. Q. J., Kruger, F. J., Layer, P. W. & Koeberl, C. The Anna's Rust Sheet and related gabbroic intrusions in the Vredefort Dome-Kibaran magmatic event on the Kaapvaal Craton and beyond? *J. African Earth Sci.* **31**, 499-521 (2000).
68. Stepto, D. The geology and gravity field in the central core of the Vredefort structure. *Tectonophysics* **171**, 75-103 (1990).
69. Stevens, G., Gibson, R. L. & Droop, G. T. R. Mid-crustal granulite facies metamorphism in the central Kaapvaal craton: The Bushveld Complex connection. *Precambrian Res.*

82, 113-132 (1997).

70. Anhaeusser, C. R. Archaean crustal evolution of the central Kaapvaal Craton, South Africa: evidence from the Johannesburg Dome. *S. Afr. J. Geol.* **102**, 303-322 (1999).
71. Anhaeusser, C. R. Metasomatized and hybrid rocks associated with a Palaeoarchaean layered ultramafic intrusion on the Johannesburg Dome, South Africa. *J. Afr. Earth Sci.* **102**, 203-217 (2015).
72. Van Tonder, D. M. & Mouri, H. Petrology and geochemistry of the granitoid rocks of the Johannesburg Dome, Central Kaapvaal Craton, South Africa. *S. Afr. J. Geol.* **113**, 257-286 (2010).
73. Hacker, B. R., Kelemen, P. B. & Behn, M. D. Continental lower crust. *Annu. Rev. Earth Pl. Sc.* **43**, 167-205 (2015).
74. Huang, Y., Chubakov, V., Mantovani, F., Rudnick, R. L. & McDonough, W. F. A reference Earth model for the heat-producing elements and associated geoneutrino flux. *Geochem. Geophys. Geosyst.* **14**, 2003-2029 (2013).
75. Moyen, J. F. The composite Archaean grey gneisses: petrological significance, and evidence for a non-unique tectonic setting for Archaean crustal growth. *Lithos* **123**, 21-36 (2011).
76. Barton Jr, J. M. The Messina layered intrusion, Limpopo Belt, South Africa: an example of in-situ contamination of an Archean anorthosite complex by continental crust. *Precambrian Res.* **78**, 139-150 (1996).
77. Barton Jr, J. M., Barton, E. S. & Kröner, A. Age and isotopic evidence for the origin of the Archaean granitoid intrusives of the Johannesburg Dome, South Africa. *J. Afr. Earth Sci.* **28**, 693-702 (1999).
78. Barton Jr, J. M., Barton, E. S. & Smith, C. B. Petrography, age and origin of the schiel alkaline complex, northern Transvaal, Southern Africa. *J. Afr. Earth Sci.* **22**, 133-145 (1996).
79. Barton Jr, J. M., Doig, R., Bohlender, F. & van Reenen, D. D. Isotopic and REE characteristics of the intrusive charnoenderbite and enderbite geographically associated with the Matok Pluton, Limpopo Belt, southern Africa. *Precambrian Res.* **55**, 451-467 (1992).
80. Barton Jr, J. M. & van Reenen, D. D. The significance of Rb-Sr ages of biotite and phlogopite for the thermal history of the Central and Southern Marginal Zone of the Limpopo Belt of southern Africa and the adjacent portions of the Kaapvaal Craton. *Precambrian Res.* **55**, 17-31 (1992).

81. Buchanan, P. C., Reimold, W. U., Koeberl, C. & Kruger, F. J. Rb-Sr and Sm-Nd isotopic compositions of the Rooiberg Group, South Africa: early Bushveld-related volcanism. *Lithos* **29**, 373-388 (2003).

82. Chavagnac, V. Behaviour of the Sm-Nd isotopic system during metamorphism: Examples from the HT-LP metamorphic terrane of the Limpopo Belt, Sourth Africa and the UHP metamorphic terrane of Dabeishan, Central China. Applied Geology. Université Rennes 1 (1999).

83. Chutas, N. I., Bates, E., Prevec, S. A., Coleman, D. S. & Boudreau, A. E. Sr and Pb isotopic disequilibrium between coexisting plagioclase and orthopyroxene in the Bushveld Complex, South Africa: microdrilling and progressive leaching evidence for sub-liquidus contamination within a crystal mush. *Contrib. Mineral. Petrol.* **163**, 653-668 (2012).

84. Curl, E. A. Parental magmas of the Bushveld Complex, South Africa. Ph.D. Thesis, Monash University, p. 164 (2001).

85. Dubinina, E. O., Aranovich, L. Y., van Reenen, D. D., Avdeenko, A. S., Varlamov, D. A., Shaposhnikov, V. V. & Kurdyukov, E. B. Involvement of fluids in the metamorphic processes within different zones of the Southern Marginal Zone of the Limpopo complex, South Africa: An oxygen isotope perspective. *Precambrian Res.* **256**, 48-61 (2015).

86. Fagereng, A., Harris, C., La Grage, M. & Stevens, G. Stable isotope study of the Archaean rocks of the Vredefort impact structure, central Kaapvaal Craton, South Africa. *Contrib. Mineral. Petrol.* **155**, 63-78 (2008).

87. Fourie, D. S. & Harris, C. O-isotope study of the Bushveld Complex granites and granophyres: constraints on source composition, and assimilation. *J. Petrol.* **52**, 2221-2242 (2011).

88. Harmer, R. E. & Sharpe, M. R. Field relations and strontium isotope systematics of the marginal rocks of the Eastern Bushveld Complex. *Econ. Geol.* **80**, 813-837 (1985).

89. Harris, C. & Chaumba, J. B. Crustal contamination and fluid-rock interaction during the formation of Platreef, Northern Limb of the Bushveld Complex, South Africa. *J. Petrol.* **42**, 1321-1347 (2001).

90. Harris, C., Fourie, D. S. & Fagereng, A. Stable isotope evidence for impact-related pseudotachylite formation at Vredefort by local melting of dry rocks. *S. Afr. J. Geol.* **116**, 101-118 (2013).

91. Harris, C., Pronost, J. M., Ashwal, L. D. & Cawthorn, R. G. Oxygen and hydrogen isotope

stratigraphy of the Rustenburg Layered Suite, Bushveld Complex: Constraints on crustal contamination. *J. Petrol.* **46**, 579-601 (2005).

92. Harris, N. B. W., Hawkesworth, C. J., Calsteren, P. V. & McDermott, F., 1987. Evolution of continental crust in southern Africa. *Earth Planet. Sci. Lett.* **83**, 85-93 (1987).
93. Hart, R. J., Andreoli, M. A. G., Smith, C. B., Otter, M. L. & Durrheim, R. Ultramafic rocks in the centre of the Vredefort structure (South Africa): Possible exposure of the upper mantle? *Chem. Geol.* **83**, 233-248 (1990).
94. Hoernes, S., Lichtenstein, U., van Reenen, D. D. & Mokgatha, K. Whole-rock/mineral O-isotope fractionations as a tool to model fluid-rock interaction in deep seated shear zones of the Southern Marginal Zone of the Limpopo Belt, South Africa. *S. Afr. J. Geol.* **98**, 488-497 (1995).
95. Hoernes, S. & van Reenen, D. D. The oxygen-isotopic composition of granulites and retrogressed granulites from the Limpopo Belt as a monitor of fluid-rock interaction. *Precambrian Res.* **55**, 353-364 (1992).
96. Huang, Y. M., van Calsteren, P. & Hawkesworth, C. J. The evolution of the lithosphere in southern Africa: A perspective on the basic granulite xenoliths from kimberlites in the South Africa. *Geochim. Cosmochim. Acta* **59**, 4905-4920 (1995).
97. Johnson, T. E., Gibson, R. L., Brown, M., Buick, I. S. & Cartwright, I. Partial melting of metapelitic rocks beneath the Bushveld Complex, South Africa. *J. Petrol.* **44**, 789-813 (2003).
98. Karykowski, B. T., Yang, S. H., Maier, W. D., Lahaye, Y., Lissenberg, C. J. & O'Brien, H. In situ Sr isotope compositions of plagioclase from a complete stratigraphic profile of the Bushveld Complex, South Africa: evidence for extensive magma mixing and percolation. *J. Petrol.* **58**, 2285-2308 (2017).
99. Kruger, F. J. Filling the Bushveld Complex magma chamber: lateral expansion, roof and floor interaction, magmatic unconformities, and the formation of giant chromitite, PGE and Ti-V-magnetitite deposits. *Mineral. Deposita* **40**, 451-472 (2005).
100. La Grange, M. A detailed petrographic and oxygen isotope study of metamorphic rocks and Archean basement complex gneisses from the Vredefort Dome, South Africa. MSc. Dissertation, University of Cape Town, South Africa., p. 124 (2004).
101. Maier, W. D., Arndt, N. T. & Curl, E. A. Progressive crustal contamination of the Bushveld Complex: evidence from Nd isotopic analyses of the cumulate rocks. *Contrib. Mineral. Petrol.* **140**, 316-327 (2000).
102. Mangwegape, M., Roelofse, F., Mock, T. & Carlson, R. W. The Sr-isotopic stratigraphy

of the Northern Limb of the Bushveld Complex, South Africa. *J. Afr. Earth Sci.* **113**, 95-100 (2016).
103. Othman, D. B., Polvé, M. & Allègre, C. J. Nd-Sr isotopic composition of granulites and constraints on the evolution of the lower continental crust. *Nature* **307**, 510-515 (1987).
104. Prevec, S. A., Ashwal, L. D. & Mkaza, M. S. Mineral disequilibrium in the Merensky Reef, western Bushveld Complex, South Africa: new Sm-Nd isotopic evidence. *Contrib. Mineral. Petrol.* **149**, 306-315 (2005).
105. Pronost, J., Harris, C. & Pin, C. Relationship between footwall composition, crustal contamination, and fluid-rock interaction in the Platreef, Bushveld Complex, South Africa. *Mineral. Deposita* **43**, 825-848 (2008).
106. Roelofse, F., Ashwal, L. D. & Romer, R. L. Multiple, isotopically heterogeneous plagioclase populations in Bushveld Complex suggest mush intrusion. *Chemie der Erde* **75**, 357-364 (2015).
107. Reimold, W. U., Hauser, N., Hansen, B. T., Thirlwall, M. & Hoffmann, M. The impact pseudotachylitic breccia controversy: Insights from first isotope analysis of Vredefort impact-generated melt rocks. *Geochim. Cosmochim. Acta* **214**, 266-281 (2017).
108. Schannor, M., Veksler, I. V., Hecht, L., Harris, C., Romer, R. L. & Manyeruke, T. D. Small-scale Sr and O isotope variations through the UG2 in the eastern Bushveld Complex: The role of crustal fluids. *Chem. Geol.* **485**, 100-112 (2018).
109. Schiffries, C. M. & Rye, D. M. Stable isotopic systematics of the Bushveld Complex: I. constraints of magmatic processes in layered intrusions. *Am. J. Sci.* **289**, 841-873 (1989).
110. Schmitz, M. D. & Bowring, S. A. Ultrahigh-temperature metamorphism in the lower crust during Neoarchean Ventersdorp rifting and magmatism, Kaapvaal Craton, southern Africa. *Geol. Soc. Am. Bull.* **115**, 533-548 (2003).
111. Schmitz, M. D., Vervoort, J. D., Bowring, S. A. & Patchett, P. J. Decoupling of the Lu-Hf and Sm-Nd isotope systems during the evolution of granulitic lower crust beneath southern Africa. *Geology* **32**, 405-408 (2004).
112. Vennemann, T. W. & Smith, H. S. Stable isotope profile across the orthoamphibole isograd in the Southern Marginal Zone of the Limpopo Belt, South Africa. *Precambrian Res.* **55**, 365-397 (1992).
113. Wilson, A. H., Zeh, A. & Gerdes, A. In situ Sr isotopes in plagioclase and traec element systematics in the lowest part of the Eastern Bushveld Complex: dynamic processes in an evolving magma chamber. *J. Petrol.* **58**, 327-360 (2017).
114. Yang, S. H., Maier, W. D., Lahaye, Y. & O'Brien, H. Strontium isotope disequilibrium of

plagioclase in the Upper Critical Zone of the Bushveld Complex: evidence for mixing of crystal slurries. *Contrib. Mineral. Petrol.* **166**, 959-974 (2013).

115. Barnes, S. J., Maier, W. D. & Curl, E. A. Composition of the marginal rocks and sills of the Rustenburg Layered Suite, Bushveld Complex, South Africa: Implications for the formation of the platinum-group element deposits. *Econ. Geol.* **105**, 1491-1511 (2010).
