# Supplementary material for: The Rustenburg Layered Suite formed as a stack of mush with transient magma chambers
Source: Nat Commun. 2021 Jan 21;12:505. doi: 10.1038/s41467-020-20778-w (PMC7820422; doi:10.1038/s41467-020-20778-w)
Supplement: Supplementary file 3 — Description of Additional Supplementary Files [file 41467_2020_20778_MOESM3_ESM.pdf]

Description of Additional Supplementary Files

File Name: Supplementary Data 1
Description: AlphaMELTS output files documenting the results of thermodynamic models of assimilation and crystallization of the Rustenburg Layered Suite.
